# Ferroptosis and its emerging role in kidney stone formation

**DOI:** 10.1007/s11033-024-09259-1

**Published:** 2024-02-20

**Authors:** Junyi Yang, Weisong Wu, Yirixiatijiang Amier, Xianmiao Li, Wenlong Wan, Yang Xun, Xiao Yu

**Affiliations:** 1 Department of Urology, Institute of Urology, Tongji Medical College, Tongji Hospital, Huazhong University of Science and Technology, Wuhan, 430030 China; 2 Department of Urology, Tongji Medical College, Tongji Hospital, Huazhong University of Science and Technology, Liberalization Ave, No. 1095, Wuhan, 430030 China

**Keywords:** Ferroptosis, Kidney stone, Lipid peroxidation, Oxidative stress

## Abstract

Kidney stone is a common and highly recurrent disease in urology, and its pathogenesis is associated with various factors. However, its precise pathogenesis is still unknown. Ferroptosis describes a form of regulated cell death that is driven by unrestricted lipid peroxidation, which does not require the activation of caspase and can be suppressed by iron chelators, lipophilic antioxidants, inhibitors of lipid peroxidation, and depletion of polyunsaturated fatty acids. Recent studies have shown that ferroptosis plays a crucial role in kidney stone formation. An increasing number of studies have shown that calcium oxalate, urate, phosphate, and selenium deficiency induce ferroptosis and promote kidney stone formation through mechanisms such as oxidative stress, endoplasmic reticulum stress, and autophagy. We also offered a new direction for the downstream mechanism of ferroptosis in kidney stone formation based on the “death wave” phenomenon. We reviewed the emerging role of ferroptosis in kidney stone formation and provided new ideas for the future treatment and prevention of kidney stones.

## Introduction

 Kidney stone is a disease with a long history. The incidence and prevalence of kidney stones have consistently risen over the last half-century, and this trend is predicted to continue. Factors such as changes in lifestyle, dietary habits, and global warming contribute to the upward trend. In accordance with the clinical guidelines of the American College of Physicians, the prevalence of kidney stones was 13% and 7% in men and women, respectively [1]. Kidney stone is prone to recurrence with a recurrence rate of 50% in 5–10 years and 75% in 20 years [2]. Over the past few decades, the mechanism of kidney stone formation has been continuously explored, including urinary supersaturation and crystallization, Randall’s plaques, sex hormones, microbiome, and immune response [3]. However, the high incidence and recurrence rate of kidney stones remain unsolved, which motivates us to continue researching the mechanism behind kidney stone formation [4].

The term “ferroptosis” was coined in 2012 to describe the form of cell death occurring in cancer cells expressing mutant *RAS* oncogene induced by the small molecule erastin [5]. Ferroptosis describes a form of regulated cell death that is driven by unrestricted lipid peroxidation, which does not require the activation of caspase and can be suppressed by iron chelators, lipophilic antioxidants, inhibitors of lipid peroxidation, and depletion of polyunsaturated fatty acids (PUFAs) [6, 7]. Under the excessive accumulation of reactive oxygen species (ROS), over-oxidized PUFA-containing phospholipids (PUFA-PLs) alter the membrane structure and increase its permeability, eventually leading to plasma membrane rupture and cell damage [8].

Previous studies on ferroptosis have found that ferroptosis is involved in a variety of pathological processes, such as tumors [9, 10], ischemia-reperfusion injury [11, 12], and other degenerative diseases [13] associated with extensive lipid peroxidation. In terms of kidney diseases, we observed that ferroptosis played an important role in acute kidney injury, renal ischemia-reperfusion injury, diabetic nephropathy, and renal cell carcinoma [14]. This inspired us to explore whether it also plays a role in kidney stone formation. Therefore, we analyzed the previous relevant studies and reviewed the potential mechanism of ferroptosis in the occurrence and development of kidney stones.

## Characteristics of ferroptosis

Ferroptosis is an iron-dependent form of non-apoptotic cell death, and it has some unique characteristics. Morphologically, it is mainly manifested as ultrastructural changes of mitochondria, such as shrinkage of mitochondria, increased density of mitochondrial membrane, and rupture of mitochondrial cristae and outer membrane. However, the cells have normal nucleus morphology [15]. Biochemically, cells undergoing ferroptosis exhibit reduced activity of System X_c_^−^ (a cysteine/glutamate antiporter system) and decreased glutathione (GSH) and glutathione peroxidase 4 (GPX4). In addition, excessive accumulation of ROS and lipid peroxides and abnormal iron metabolism are also important biochemical features of ferroptosis [16]. However, a recent study found that strong lipid peroxidation also occurs in atypical pyroptosis, so it should no longer be used as the sole biomarker for validating ferroptosis [17].

## The main regulatory mechanisms of ferroptosis

**Fig. 1:**
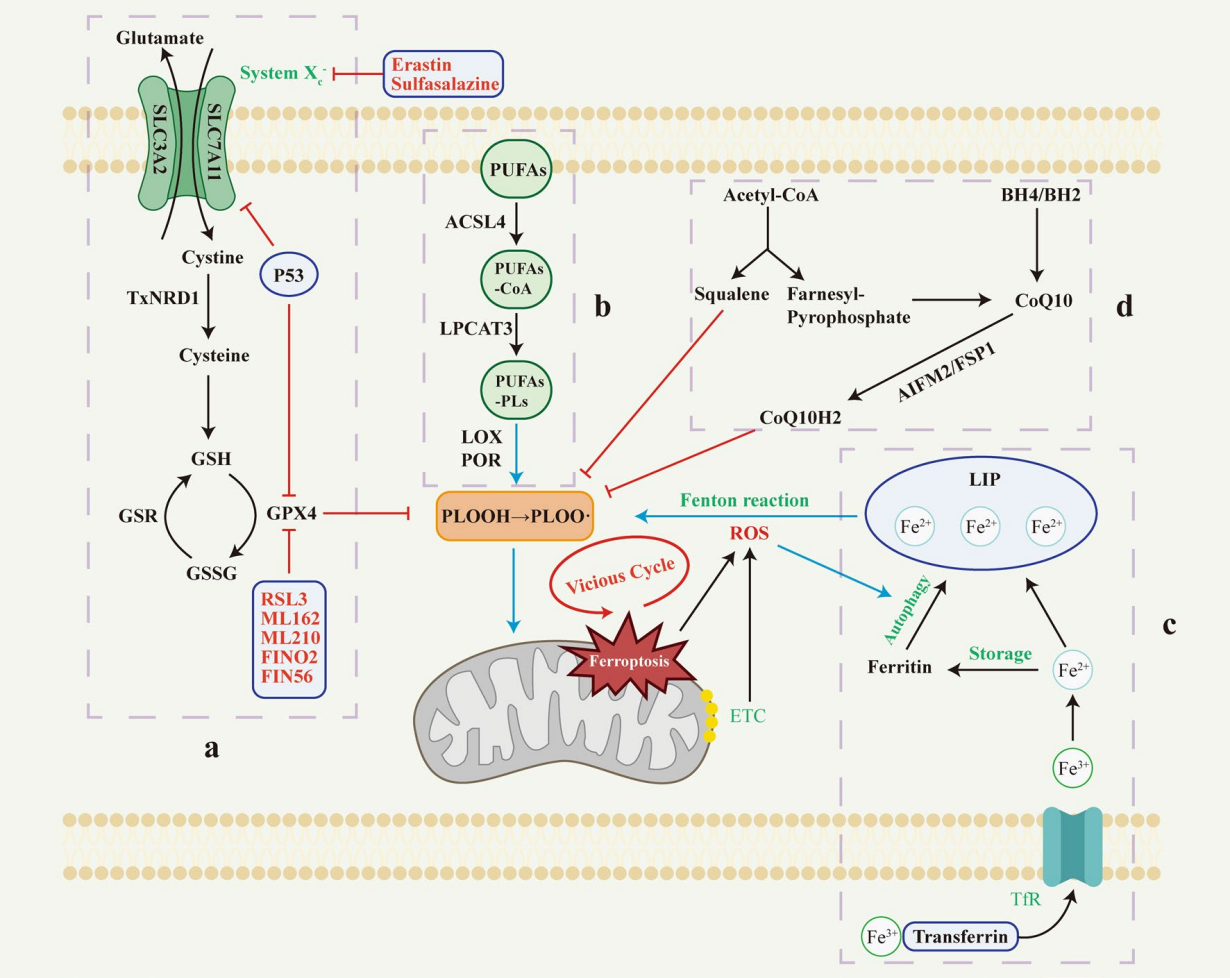
Regulatory mechanism diagram of ferroptosis. The main mechanisms regulating ferroptosis include (1) the antioxidant pathway of GPX4, (2) iron metabolism involving autophagy and Fenton reaction, (3) lipid metabolism involving enzymatic reactions, and (4) CoQ antioxidant activity. *ACSL4* acyl‐CoA synthetase long‐chain family member 4, *AIFM2* Apoptosis-inducing factor mitochondria-associated 2, *BH2* dihydrobiopterin, *BH4* tetrahydrobiopterin, *CoA* coenzyme A, *CoQ10* coenzyme Q10, *CoQ10H2* ubiquinol, *ETC* electron transport chains, *FSP1* ferroptosis suppressor protein 1, *GPX4* glutathione peroxide 4, *GSH* glutathione, *GSR* glutathione-disulfide reductase, *GSSG* glutathione disulfide, *LIP* labile iron pool, *LOX* lipoxygenase, *LPCAT3* lysophosphatidylcholine acyltransferase 3, *PLOOH* phospholipid hydroperoxides, *POR* cytochrome P450 oxidoreductase, *PUFAs* polyunsaturated fatty acids, *PUFAs-PLs* PUFA-containing phospholipids, *ROS* reactive oxygen species, *SLC3A2* solute carrier family 3 member 2, *SLC7A11* solute carrier family 7 member 11, *TfR* transferrin receptor, *TXNRD1* thioredoxin reductase 1

 Since ferroptosis was coined, the underlying mechanism has been continuously explored. Previous studies have reported that ferroptosis is regulated by multiple metabolic and signaling pathways, and we summarized the roles of these pathways in ferroptosis. (Fig. 1)

### System X_c_^−^ /GSH/GPX4 pathway

Reduced glutathione (GSH) is an important antioxidant and free radical scavenger synthesized from glutamate, cysteine, and glycine [18]. It is a cofactor used by glutathione peroxidase 4 (GPX4) to eliminate lipid peroxides in the cell membranes [19]. GPX4 is a selenocysteine (Sec)-containing antioxidant enzyme that is the only enzyme that reduces phospholipid peroxide [20, 21]. The content of GSH and nicotinamide adenine dinucleotide phosphate (NADPH) directly affects the GPX4 activity [22]. As one of the synthetic sources of GSH, cysteine is mainly derived from the System X_c_^−^ pathway and the transsulfuration pathway. System X_c_^−^ is a transmembrane transporter of cystine and glutamic acid, composed of solute carrier family 7 member 11 (SLC7A11) and solute carrier family 3 member 2 (SLC3A2) [23]. It transfers cystine or cystine sulfide into cells and reduces it to cysteine without the involvement of ATP [24]. Therefore, either inhibition of System X_c_^−^ to reduce the GPX4 enzyme activity by GSH depletion or direct inhibition of GPX4 activity can induce ferroptosis. Some classical ferroptosis inducers can generally be divided into two categories, one is to inhibit System X_c_^−^, such as sorafenib, sulfasalazine, and erastin. However, the latest research shows that sorafenib does not trigger ferroptosis through inhibition of System X_c_^−^ [25]. The other is to directly inhibit GPX4 activity, such as RSL3 [26], ML162 [27], ML210 [28], FINO2 [29], and FIN56 [30].

### Lipid peroxidation pathway

The process by which PUFA-rich membrane structures are damaged by ROS and affect membrane function is known as lipid peroxidation. ROS are formed as a result of incomplete reduction of oxygen and can be produced by mitochondrial electron transport chains (ETC) and enzymatic reactions such as NADPH oxidases (NOXs) [31]. Oxidation of free PUFA containing arachidonic acid and adrenergic acid is a prerequisite for the transmission of ferroptosis signals [32]. Acyl-CoA synthetase long-chain family member 4 (ACSL4) can bind free PUFAs to coenzyme A [33]. These products are then re-esterified into phospholipids by lysophosphatidylcholine acyltransferase 3 (LPCAT3), thereby affecting the transmembrane properties of PUFAs and allowing their incorporation into cell membranes and lipids [34]. Lipid peroxidation can be triggered by non-enzymatic and enzymatic mechanisms. The non-enzymatic mechanism is driven by the Fenton reaction, which utilizes iron and oxygen to catalyze a chain reaction, leading to the formation of phospholipid hydroperoxides [35]. The enzymatic mechanism mainly involves lipoxygenase (LOX) and cytochrome P450 oxidoreductase (POR). LOX is a non-heme iron-dependent dioxygenase that can directly oxidize PUFA and PUFA-PLs in membranes, leading to ferroptosis [36]. Although there is evidence that LOX may not be a key driver of ferroptosis, it may contribute to the initiation or propagation of injury [37]. POR can generate ROS by extracting hydrogen from PUFAs or reducing Fe^3+^ to Fe^2+^, reacting with adjacent lipids to form lipid hydroperoxides [38].

### Iron metabolism

Iron is one of the important metal ions involved in human metabolism and plays a crucial role in promoting ferroptosis. Fe^2+^ formed by intestinal absorption or erythrocyte degradation can be oxidized by copper cyanide to Fe^3+^, which binds to transferrin on the cell membrane and is further transported into the cell via transferrin receptor 1 (TFR1) [39]. In cells, Fe^3+^ is reduced again to Fe^2+^ by ferric reductase, and Fe^2+^ is released into the labile iron pool (LIP) [32]. The excess Fe^2+^ is stored in ferritin or metabolized in vivo. Once excess, Fe^2+^ can react with hydrogen peroxide in the Fenton reaction to produce ROS, ultimately leading to ferroptosis. Previous studies have found that some molecules can promote or inhibit ferroptosis by regulating iron metabolism. Nuclear receptor coactivator 4 (NCOA4) mediates ferritin degradation, causing iron release, and heme oxygenase-1 (HO-1) can catalyze the degradation of heme to produce iron to LIP [40]. When ferritinophagy activation and/or HO-1 overexpression increase free iron levels, it leads to an accumulation of lipid peroxides and ferroptosis. However, the promotion or inhibition of HO-1 for ferroptosis has obtained contradictory results, so the relationship between them needs to be further explored [41, 42]. In contrast, heat shock protein B1 (HSPB1) counteracts the increased expression of TFR1 and thus reduces the intracellular iron to inhibit ferroptosis [43].

### CoQ system

Acetyl-CoA synthesizes steroids (squalene) and precursors of CoQ (farnesyl pyrophosphate) via the mevalonate (MVA) metabolic pathway [37, 44]. A study on cholesterol auxotrophic lymphomas found that squalene alters the cellular lipid profile and protects cancer cells from ferroptosis [45]. CoQ10 is the most common form of CoQ (ubiquinone) used as a dietary supplement, and the reduced form of CoQ10, ubiquinol (CoQ10H2), is an endogenous antioxidant for cells. It has antioxidant effects on cell membranes and inhibits ferroptosis by blocking the process of lipid peroxidation [31]. GTP cyclic hydrolase 1 (GCH1) synthesizes tetrahydrobiopterin (BH4) and dihydrobiopterin (BH2), of which BH4 has been shown to act as a direct radical-trapping antioxidant and to participate in ubiquinone synthesis to inhibit ferroptosis [46, 47]. Apoptosis-inducing factor mitochondria-associated 2 (AIFM2)/ferroptosis suppressor protein 1 (FSP1) is a key component of the CoQ antioxidant system. They can reduce CoQ10 to CoQ10H2 and effectively mitigate ferroptosis by neutralizing the accumulation of lipid peroxides [48, 49].

### Others

In recent years, research on the mechanism of ferroptosis has grown rapidly, including some more discussed mechanisms such as the nuclear factor erythroid 2-related factor 2 (Nrf2), thioredoxin, p53, and NOXs. Nrf2 is a crucial oxidative-stress-responsive transcription factor that governs the expression of antioxidants and cytoprotective genes, protecting cells against oxidative stress to maintain cellular ROS homeostasis [50]. Thioredoxin can react with ROS and compensate for reduced GSH levels to maintain a reduced state of proteins [51, 52]. The TP53 gene is an essential tumor suppressor gene for humans. Inhibition of cysteine uptake by p53 can sensitize cells to ferroptosis through downregulating the expression of SLC7A11 and GPX4, reduction of antioxidant capacity, and resultant ROS accumulation [53, 54]. NOX-mediated bio-oxidation is a significant pathway for lipid-free radical production. Overexpression of NOXs can deplete NADPH and elevate oxidative free radical levels, which significantly increases the sensitivity of cells to ferroptosis [44].

## Ferroptosis and kidney stone

### Whether ferroptosis relates to kidney stones?

The formation of kidney stones originates from damage to renal tubular epithelial cells (RTECs). In the past decades, most studies on the mode of cell injury have been limited to necrosis and apoptosis. In recent years, with the progressive understanding of ferroptosis, several studies have demonstrated a correlation between ferroptosis and kidney stones. Yang et al. [55] established a rat kidney stone model by injecting glyoxalate and found that the mitochondria in rat RTECs became smaller and increased in membrane density, and the mitochondria were severely damaged. The change of mitochondrial ultrastructure was the morphological characteristic of ferroptosis in the cells, which proved that ferroptosis was involved in the formation of kidney stones. In addition, some studies have found significantly higher levels of thiobarbituric acid reactive substances (TBARS, a marker of lipid peroxidation) as well as malondialdehyde (MDA, a marker of cellular oxidative damage) in the urine of patients with kidney stones [56, 57]. A significant decrease in the activity of antioxidant enzymes such as SOD and GPX was also observed in patients with kidney stones [58]. All these findings also suggest that ferroptosis is associated with kidney stones, as both lipid peroxidation and reduced antioxidant enzyme activity are biochemical features of ferroptosis. Xie et al. [59] reported that Ferrostatin-1 (a ferroptosis inhibitor) was able to reduce calcium oxalate (CaOx)-induced RTECs injury both in vitro and in vivo. What’s more, Randall’s plaque, as the central mechanism of kidney stone formation, is thought to be a form of ectopic calcification. Coronary atherosclerosis, another ectopic calcification disease, has been shown to occur in association with ferroptosis [60]. Thus, we speculate that kidney stone, an ectopic calcification that occurs on the renal papilla, may also be associated with ferroptosis.

### What triggers ferroptosis related to kidney stones?

#### Calcium oxalate

CaOx crystal deposition is one of the important steps in the formation of CaOx stones. Previous studies have demonstrated that CaOx is an important risk factor for inducing ferroptosis. He et al. [61] reported a significant increase in the expression of ferroptosis agonist protein and a significant decrease in the expression of ferroptosis inhibitor protein, as well as a significant increase in MDA levels in HK-2 cells with increasing CaOx concentration. Song et al. [62] found rupture of the outer mitochondrial membrane and disappearance of mitochondrial cristae in oxalate-treated HK-2 cells. This proved that CaOx activated ferroptosis in HK-2 cells in a concentration-dependent manner. In addition, Kang et al. [63] found that ethylene glycol-induced CaOx crystals in rats induced autophagy and upregulated the expression of BECN1. BECN1 not only regulates autophagy but also affects cystine uptake by binding to System X_c_^−^, leading to inhibition of GPX4 and increased levels of lipid peroxidation, inducing ferroptosis [64, 65]. Song et al. [62] similarly observed the occurrence of autophagy and a significant increase in BECN1 protein levels in oxalate-treated HK-2 cells. The knockdown of BECN1 in HK-2 cells resulted in a significant decrease in lipid peroxide levels, a significant decrease in ROS levels, and a significant increase in GPX4 levels. This demonstrated that CaOx could induce ferroptosis by activating autophagy and upregulating BECN1. What’s more, Mulay et al. [66] found that CaOx crystals could drive peptidylprolyl isomerase F, promoting the opening of mitochondrial permeability transition pores. This mechanism drives the breakdown of the mitochondrial outer membrane potential, mitochondrial swelling, loss of cristae, and massive ROS production. CaOx also upregulates the renin/angiotensin system, activating NOXs in RTECs and exacerbating ROS production [67, 68]. These factors lead to excessive accumulation of ROS and induce lipid peroxidation, promoting the development of ferroptosis.

#### Urine microenvironment

In addition to CaOx crystals, a few substances that may affect the urinary microenvironment have been reported to be strongly associated with ferroptosis, such as high uric acid, high calcium, and high phosphate. Ye et al. [69] found that high concentrations of calcium and phosphate were able to inhibit the SLC7A11/GSH/GPX4 pathway in rat vascular smooth muscle cells, thereby inducing ferroptosis. Yu et al. [70] reported that high uric acid can downregulate the Nrf2/SLC7A11/GPX4 pathway to induce ferroptosis in macrophages, which is involved in the formation of atherosclerotic plaques. Although hypocitraturia is also an important risk factor for kidney stones, no study has reported its correlation with ferroptosis. However, little research has been done in this area. Whether they can be involved in the formation of kidney stones by inducing ferroptosis still needs to be further explored.

#### Selenium

Selenium is an essential non-metallic element with antioxidant, anti-inflammatory, and immunomodulatory properties in the human body [71]. A recent cross-sectional study found that dietary selenium intake was negatively associated with the incidence of kidney stones in adults [72]. Several studies have previously reported the inhibitory effect of selenium on kidney stones. Sakley et al. [73] first reported that intraperitoneal administration of selenium inhibited CaOx deposition in rats. A study in dogs found that selenium reduced glycolysis-induced CaOx stones by inhibiting osteopontin expression [74]. However, the mechanism by which selenium inhibits the development of kidney stones remains unclear. As we all know, among the 25 selenoproteins that have been identified in humans, both GPXs and thioredoxin reductases (TXNRDs) are involved in the antioxidant function of cells, and both are involved in the occurrence of ferroptosis [75, 76]. Selenium is not only a component of GPX4 but also drives GPX4 transcription and regulates the antioxidant machinery by coordinating the activation of transcription factors transcription factor activating protein 2 gamma (TFAP2c) and Sp1 [77]. In addition, several studies have demonstrated the protective effect of selenium against ferroptosis. Ingold et al. [78] found that cells were highly sensitive to peroxide-induced ferroptosis by replacing selenocysteine in GPX4 with cysteine, demonstrating the importance of selenium for GPX4 to function as an antioxidant against ferroptosis. Shi et al. [79] reported that selenium could upregulate mitofusin-1 (Mfn1) expression to promote mitochondrial fusion, thereby alleviating ferroptosis and attenuating brain ischemia-reperfusion injury in mice. Wu et al. [80] found that selenium could inhibit ferroptosis through the Nrf2/GPX4 signaling pathway and ameliorate autistic-like behaviors in mice. We therefore speculate that selenium may be a protective factor against ferroptosis and thus inhibit the development of kidney stones.

### How to trigger ferroptosis?

As mentioned above, both phosphate and urate can downregulate GPX4 expression and reduce the antioxidant capacity of cells. CaOx crystals can activate NOXs to release large amounts of ROS. These factors together lead to the occurrence of oxidative stress in cells. Under oxidative stress, excessive ROS emissions lead to mitochondrial damage, and loss or defects in mitochondrial energy production can enhance metabolic processes in the tricarboxylic acid (TCA) cycle, ETC, exacerbating ROS release and reducing antioxidant enzymes [81–83]. At high concentrations of ROS, lipids, especially PUFAs, are oxidatively damaged, causing lipid peroxidation and inducing ferroptosis. In addition, Park et al. [84] reported that ROS can mediate ferritin autophagy and upregulate TFR1 expression, promoting intracellular iron overload and ferroptosis.

In addition to damaging lipids, large amounts of ROS disrupt endoplasmic reticulum homeostasis and lead to the accumulation of misfolded and unfolded proteins, activating unfolded protein responses and triggering endoplasmic reticulum stress (ERS) [85]. Zhang et al. [86]. found that selenium could protect mouse kidneys from apoptosis and oxidative stress by inhibiting ERS in a mouse kidney injury model, suggesting that selenium may mitigate ferroptosis by inhibiting the ERS pathway. Wang et al. [87] found that artemisinin could activate the ATF4/CHOP/CHAC1 pathway in ERS to block GSH synthesis, ultimately inducing ferroptosis in Burkitt’s lymphoma. Previous studies have found that ERS is associated with the development of CaOx kidney stones [88, 89]. This evidence suggests that ERS may influence the development of stones through ferroptosis. (Table 1).

**Table 1:** Functions of factors of kidney stone and their promotion or resistance of ferroptosis

Factor	Function	Promote or resist ferroptosis	Reference(s)
CaOx	Activate autophagy and upregulating BECN1	Promote	[62, 63]
	Drive Ppif and promote the opening of MPT pores		[66]
	Upregulates the renin/angiotensin system		[67, 68]
Phosphate	Inhibit the SLC7A11/GSH/GPX4 pathway	Promote	[69]
Urate	Downregulate the Nrf2/SLC7A11/GPX4 pathway	Promote	[70]
Se	Drives GPX4 transcription and regulates the antioxidant machinery by coordinating the activation of transcription factors TFAP2c and Sp1	Resist	[77]
	Synthetic GPX4		[78]
	Upregulate Mfn1 expression		[79]
	Upregulate the Nrf2/GPX4 signaling pathway		[80]
	Inhibit endoplasmic reticulum stress		[86]

*BECN1* beclin-1, *CaOx* calcium oxalate, *GPX4* glutathione peroxide 4, *GSH* glutathione, *MPT* mitochondrial permeability transition, *Nrf2* nuclear factor erythroid 2-related factor 2, *Ppif* peptidylprolyl isomerase F, *Se* selenium, *SLC7A11* solute carrier family 7 member 11

Autophagy is a process by which cells in vivo produce autophagic vesicles to engulf damaged organelles and macromolecules in the presence of nutrient deficiency or oxidative stress and use lysosomes to degrade them, thereby maintaining the stability of the intracellular environment [90]. More and more evidence shows a close relationship between ERS and autophagy, with autophagy being an important downstream event of ERS [91]. Excessive activation of autophagy will disrupt intracellular environmental homeostasis [92]. Several studies have found that cadmium can promote ferroptosis through ERS-mediated activation of autophagy, revealing a potential mechanism for ferroptosis activation by the ERS/autophagy axis [93, 94]. As an upstream event of ferroptosis, autophagy activation can induce ferroptosis through different mechanisms, including NCOA4-mediated ferritin autophagy [95], RAB7A-mediated lipid autophagy [96], ARNTL-mediated biological clock protein autophagy [97], and BECN1-mediated System X_c_^−^ inhibition [64]. We mentioned earlier that CaOx crystals can induce ferroptosis by activating autophagy and upregulating the expression of BECN1. This suggests that CaOx crystals may induce ferroptosis through autophagy and promote kidney stone development.

### How does ferroptosis mediate kidney stone formation?

**Fig. 2:**
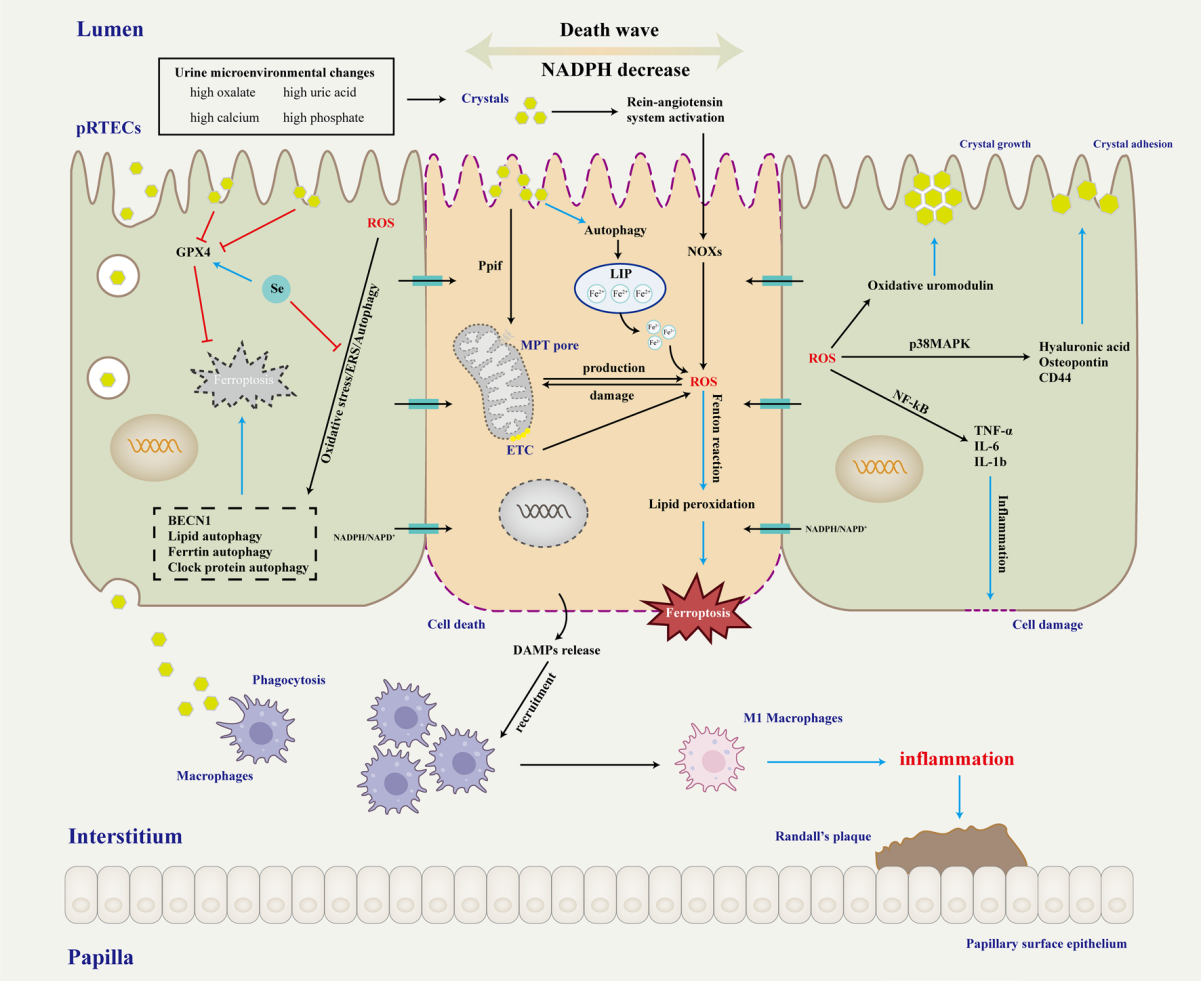
Mechanism of ferroptosis in pRTECs and kidney stone information. Supersaturation of chemicals in urine forms crystals and triggers mitochondrial damage and ROS accumulation in RTECs, ultimately inducing ferroptosis. Through free diffusion of NADPH, the dead cells reduce the antioxidant capacity of neighboring cells, causing oxidative stress and possibly inducing ferroptosis in neighboring cells, forming the “death wave” phenomenon. The accumulation of ROS in neighboring cells can cause damage to RTECs and promote crystal adhesion and aggregation. The dead cells undergo plasma membrane rupture and release DAMPs, which activate and recruit macrophages. Macrophages can polarize toward the M1 type to promote inflammatory responses, which ultimately provide the conditions for stone formation. *BECN1* beclin-1, *CD44* Cluster of differentiation-44, *DAMPs* damage-associated molecular patterns, *ERS* endoplasmic reticulum stress, *ETC* electron transport chains, *GPX4* glutathione peroxide 4, *IL* interleukin, *LIP* labile iron pool, *MPT* mitochondrial permeability transition, *NADPH* nicotinamide adenine dinucleotide phosphate, *NF-kB* nuclear transcription factor-kappa B, *NOXs* NADPH oxidases, *p38MAPK* p38 mitogen activated protein kinase, *Ppif* peptidylprolyl isomerase F, *ROS* reactive oxygen species, *pRTECs* proximal renal tubular epithelial cells, *Se* selenium, *TNF-α* tumor necrosis factor-α

Most of the current studies have focused on the upstream mechanism by which risk factors for kidney stones induce ferroptosis in RTECs. However, what happens after the cell undergoes ferroptosis to lead to stone formation? This part is currently lacking, hence our conjecture with a view to providing ideas for further research. (Fig. 2).

A recent genome-wide association study showed that the key target tissues and cell types of kidney stones are mainly proximal renal tubules, and proximal renal tubular epithelial cells (pRTECs) injury further promotes kidney stone formation [98]. Therefore, the initiating cellular abnormality causing kidney stone formation may occur in pRTECs. Currently, the pathogenesis of kidney stones is mainly based on the “Randall plaque” doctrine and the “Randall plug” doctrine [99]. The “Randall plug” doctrine suggests that crystals nucleate, grow, and aggregate in the urine of the renal tubules. The crystal deposits block the Bellini tubules forming Randall's plug. The plug protrudes into the renal pelvis and is exposed to the urine, further promoting the formation of small stones behind the plug. This doctrine is also the basis for most current cellular or animal models of kidney stones. The “Randall plaque” doctrine suggests that kidney stones begin as calcium plaques on the basement membrane of the loop of Henle, and that large numbers of idiopathic CaOx stones attach to the plaques to form deposits deep in the renal interstitium. Its formation may be related to collagen mineralization. It is well known that pRTECs have abundant microvilli on the luminal side of the tubule, which influences the fluid flow rate and the movement of crystals. Smaller crystals can be retained by attachment of microvilli to the epithelial surface [100]. Crystal deposition leads to epithelial damage and cellular ferroptosis. The death of one cell in the epithelial layer of the renal tubule may initiate a chain reaction [101]. pRTECs are interconnected by gap junctions and tight junctions, and when ferroptosis occurs in one cell, NADPH can simply diffuse from adjacent cells to the dead cell via cell junctions, thus making the adjacent cells less antioxidant and more sensitive to ferroptosis [101, 102]. Oxidative stress causes pRTECs to overaccumulate ROS and damage mitochondria, exacerbating oxidative damage. The role of oxidative stress in stone formation has been widely reported [103–106] (Table 2).

When oxidative damage accumulates again to a certain level, ferroptosis is also induced in these originally adjacent cells, which continues to lead to a reduction in the antioxidant capacity of surrounding cells, ultimately leading to large areas of pRTECs damage. On the one hand, dead cells or cellular debris accumulate in the tubular lumen and promote crystal aggregation. Cell degradation products become part of the organic matrix for stone growth in the renal tubules. The aggregated crystals move with the urine to the distal collecting ducts, forming "Randall's plug" and accelerating kidney stone formation. On the other hand, cell death exposes the basement membrane, to which crystals bind directly and on which regenerating epithelium grows, pushing the crystals into the interstitium and promoting crystal retention. It is possible that this further contributed to the inflammatory response and calcification, forming “Randall's plaque”. pRTECs undergo ferroptosis followed by rupture of the plasma membrane and release of damage-associated molecular patterns (DAMPs) such as high mobility group box 1 (HMGB1), ATP, DNA, mtDNA, oxidized phospholipids and MDA [107]. DAMPs usually activate the innate immune response and macrophages are recruited and activated to secrete various mediators such as macrophage inhibitory protein-1, monocyte chemoattractant protein-1, and IL-8, leading to interstitial inflammation in the kidney [108]. Khan et al [68]. demonstrated an association between inflammation and crystal deposition and Randall’s plaque formation. In addition, these chemokines secreted by macrophages enhance the recruitment of various immune cells, in which infiltrated monocytes can differentiate into different macrophage subtypes and play an important role in renal CaOx crystal formation [3]. Although cellular abnormalities in the initiating cells of kidney stone may occur in the pRTECs, both Randall's plaque and plug occur in the more distal renal tubules, and thus the links between pRTECs and more distal events remain unclear. Although much evidence suggests that ferroptosis plays a significant role between pRTECs and kidney stone, the exact mechanisms need to be further explored.

## Conclusion and prospects

The occurrence of ferroptosis can be summarized as disruption of iron metabolic homeostasis or System X_c_^−^/GSH/GPX4 pathway by various factors causing the depletion of cellular antioxidants and amplification of ROS production, ultimately leading to the accumulation of lipid peroxides and triggering ferroptosis. During the development of kidney stones, risk factors such as CaOx crystals, urine microenvironmental changes, and selenium deficiency induce ferroptosis through mechanisms such as oxidative stress, ERS, and autophagy. pRTECs that undergo ferroptosis may initiate a chain reaction that reduces the antioxidant capacity of surrounding cells, which in turn leads to oxidative damage to the renal tubules, furtherly promoting crystal aggregation and adhesion. In conclusion, risk factors for kidney stones induce ferroptosis in the epithelial cells of the proximal tubules, and the chain of occurrence further accelerates the deposition and aggregation of crystals involved in kidney stone formation.

 As a global disease, kidney stones place a financial burden on patients and put a strain on the healthcare system. With the development of minimally invasive techniques, most patients with kidney stones opt for surgical treatment. In contrast, pharmacological treatment modalities have not advanced significantly over the past 30 years, relying primarily on drugs that alter urinary chemicals to reduce crystalline salt supersaturation. The potential role of ferroptosis in kidney stones has opened our eyes to new therapeutic targets. However, as a novel cell death mechanism, the mechanism by which ferroptosis promotes stone production is currently unknown. The application of ferroptosis inhibitors to the treatment and prevention of kidney stones is challenging, and the question of whether ferroptosis inhibitors cause decreased resistance of the body to potentially malignant cells and whether they cause damage to other tissues of the kidney needs to be further explored. Therefore, we need to clarify the specific mechanism of ferroptosis inhibition of kidney stone occurrence, which is beneficial to the targeted and personalized treatment of kidney stones, providing knowledge for precision medicine and new ideas for kidney stone prevention and treatment.

**Table 2:** Functions of ROS and its risks in kidney stone

Factor	Function	Risk in kidney stone	Reference(s)
ROS	Upregulate the expression of hyaluronic acid, osteopontin and CD44 via the p38MAPK pathway	Alter the adhesion of RTECs to CaOx crystals	[103]
	Oxidize uromodulin	Promote CaOx crystallization and crystal growth	[104]
	Initiate the NF-kB signaling pathway, promoting the secretion of cytokines such as TNF-α, IL-6 and IL-1b	Lead to RTECs damage, detachment and basement membrane exposure	[105, 106]

*CaOx* calcium oxalate, *CD44* Cluster of differentiation-44, *IL* interleukin, *NF-kB* nuclear transcription factor-kappa B, *p38MAPK* p38 mitogen activated protein kinase, *ROS* reactive oxygen species, *RTECs* renal tubular epithelial cells, *TNF-α* tumor necrosis factor-α
